# Learning to Reformulate Peer Conflict: Effects of a Language-Based Educational Program on Conflict Management, Peer Interaction, and Bullying-Related Experiences in Early Adolescence

**DOI:** 10.3390/ejihpe16070093

**Published:** 2026-06-30

**Authors:** Katia Rolán, Francisca Fariña, Miguel Cuevas-Alonso

**Affiliations:** 1 Laboratorio de Linguaxe e Cognición, Universidade de Vigo, 36310 Vigo, Spain; miguel.cuevas@uvigo.gal; 2 Instituto de Investigación Lingua, Universidade de Vigo, 36310 Vigo, Spain; 3 Grupo Language Variation and Textual Categorisation (LVTC), Universidade de Vigo, 36310 Vigo, Spain; 4 Cátedra UNESCO Educación Transformadora: Ciencia, Comunicación y Sociedad, Universidade de Vigo, 36005 Pontevedra, Spain; francisca@uvigo.gal

**Keywords:** communication-based intervention, conflict management, early adolescence

## Abstract

Peer conflict in early adolescence ranges from everyday disagreements to repeated aggression and bullying-related experiences. Bullying should not be reduced to physical violence: it may involve relational exclusion, psychological harassment, or verbal aggression without direct physical harm, whereas isolated aggressive acts do not necessarily constitute bullying. This study evaluated *Reformulando*, a school-based program that trains communication-based socio-emotional skills through pragmatic reformulation and respectful language. Using a quasi-experimental pretest–posttest design with a non-equivalent control group, we assessed changes in conflict management strategies, peer aggression, and bullying-related experiences among students aged 10–13 years. Initial analyses suggested reductions in verbal peer aggression in the experimental group relative to the control group, although these effects were attenuated in age-adjusted and classroom-clustered sensitivity analyses. Collaborative conflict management increased over time, although this change did not differ significantly between groups. Bullying-related experiences decreased during the study period, but these reductions were not specific to the experimental group. This pattern emerged in the primary analyses but was attenuated in age-adjusted and cluster-adjusted sensitivity analyses. Findings suggest the potential value of brief communication-based training for addressing verbal peer conflict, while highlighting the need for cautious interpretation due to baseline group differences, grade-level imbalance, classroom clustering, and the quasi-experimental design.

## 1. Introduction

Peer conflict is common in late childhood and early adolescence and may manifest as disagreements, teasing, exclusion, or aggression. A subset of these experiences may develop into bullying, typically characterized by repetition, intentional harm, and an imbalance of power, although definitions and terminology vary across countries and developmental groups (Smith et al., 2002). Importantly, bullying should not be reduced to physical violence. It may involve relational exclusion, psychological harassment, or verbal aggression without direct physical harm, whereas isolated aggressive acts do not necessarily constitute bullying. Conflating these constructs reduces conceptual clarity, weakens measurement validity, and complicates interpretation. This conceptual caution is consistent with analyses showing that bullying and pupil violence have been framed differently across educational and policy contexts (Boge & Larsson, 2018). In this paper, we therefore distinguish between (a) peer aggression or conflict tactics (verbal/physical behaviors that may occur in conflict episodes) and (b) bullying-related experiences (recurrent harassment and exclusion measured via validated subdomains). These dynamics can negatively affect socio-emotional development, peer relationships, and classroom climate, underscoring the importance of educational approaches that foster adaptive communication and interpersonal skills. Maladaptive behavior in school is also closely related to students’ basic psychological needs and adjustment within the classroom environment (Oostdam et al., 2019). Educational interventions targeting socio-emotional competencies and positive peer interaction have become increasingly relevant in school-based prevention programs, particularly during early adolescence, a developmental stage characterized by heightened interpersonal sensitivity and social identity formation (Durlak et al., 2011; Taylor et al., 2017).

International research has documented the prevalence and health consequences of bullying and cyberbullying, including psychological distress, impaired school functioning, poorer peer adjustment, and broader psychosocial difficulties (Beaty & Alexeyev, 2008; Houbre et al., 2006; Lopes Neto, 2005; Pearce & Thompson, 1998). Beyond offline settings, digital environments have become a major context for adolescent victimization, with evidence linking cyberbullying and school bullying to psychological distress among adolescents (Schneider et al., 2012). In addition to individual vulnerabilities, school climate and peer norms shape whether conflict escalates into repeated harm (Cook et al., 2010; Thapa et al., 2013). From an educational psychology perspective, communication practices can be understood as learnable socio-emotional skills that shape how students interpret, regulate, and respond to interpersonal situations. This view is consistent with educational research showing that the quality of student dialog is relevant for citizenship education and socio-moral development (Schuitema et al., 2011).

Importantly, the words people select when narrating conflicts are not neutral. This view is consistent with evidence showing that linguistic choices in conflict narratives are closely linked to emotional intensity and meaning construction, shaping how individuals interpret and respond to interpersonal situations (Rolán et al., 2025). For example, derogatory labels, insults, and discriminatory talk can intensify negative affect and motivate retaliatory behaviors, thereby increasing the likelihood of escalation. Critical approaches to bullying and harassment have also emphasized the role of gendered, discriminatory, and exclusionary language in school contexts (Meyer, 2008).

Programs that reduce bullying and improve school coexistence typically combine multiple components, including school-wide policies, supervision, classroom rules, monitoring, skills training, and individual support strategies (Mooij, 2008; Olweus & Limber, 2010). Meta-analytic evidence suggests that school-based anti-bullying programs can reduce bullying perpetration and victimization, although effects are heterogeneous and often modest (Gaffney et al., 2021; Ttofi & Farrington, 2011). Within this broad landscape, the specific role of language-based micro-skills has received comparatively less focus. The *Reformulando* program was developed to address this gap by promoting communication-based socio-emotional competencies through pragmatic reformulation as an applied communication technique for peer conflict. Pragmatic reformulation should not be understood as simple repetition or neutral paraphrasing. In mediation, reformulation involves returning another person’s message in a modified form that preserves its core meaning while reducing accusatory framing, clarifying communicative intent, and making emotional content more explicit and manageable (Fischer-Lokou et al., 2016; Rolán, 2021). From this perspective, reformulation may operate as a language-mediated reappraisal process: by changing how a conflict is verbally represented, students may also change how they interpret the other person’s intention, the emotional meaning of the situation, and the range of possible responses available to them (Gross, 1998; Lieberman et al., 2007; Webb et al., 2012). This mechanism is especially relevant for verbal peer aggression, because insults, threats, and hostile labels are themselves linguistic acts that can intensify negative affect and promote retaliation. The expected pathway from reformulation to physical aggression or bullying-related experiences is necessarily more indirect. Reactive aggression has been linked to hostile interpretations of ambiguous peer behavior and to difficulties in generating non-aggressive responses (Crick & Dodge, 1996; Dodge, 2006). Teaching students to reformulate may therefore help slow down automatic hostile attributions, introduce alternative interpretations, and reduce verbal escalation before a conflict moves toward retaliation. However, physical harassment, exclusion, and systematic bullying also involve broader factors such as power imbalance, peer group norms, classroom climate, and repeated social positioning. For this reason, the program was expected to have its most proximal effects on verbal peer aggression, whereas possible effects on physical aggression and bullying-related experiences were considered more distal, context-dependent, and exploratory. Previous linguistic-discursive work on students’ responses to conflict situations in Primary Education provided an applied basis for the development of this program (Acuña et al., 2017). In this framework, reformulation is conceptualized not only as a linguistic strategy, but also as a communication-based socio-emotional skill that may foster emotional regulation, perspective-taking, and constructive peer interaction. Developmental timing is also relevant for interpreting the findings. Even within a relatively narrow age range, students may differ in socio-emotional maturity, peer status concerns, classroom hierarchies, and sensitivity to social evaluation (Steinberg & Morris, 2001; Eccles & Roeser, 2011; Yeager et al., 2018). These differences may influence how students respond to communication-based training: younger students may benefit more from explicit modeling of reformulation and respectful language, whereas older students may be more influenced by established peer norms and status dynamics. Because the study used intact classrooms and age/grade were not experimentally manipulated, we did not formulate a directional age- or grade-moderation hypothesis. Age and grade-level composition were therefore treated as relevant contextual features and potential sources of non-equivalence between groups.

The objective of this study was to evaluate the educational and socio-emotional impact of the *Reformulando* program on conflict management, peer interaction, and bullying-related experiences in early adolescence.

We tested the following hypotheses:

**H1 (Conflict resolution styles):**  *Students receiving the program will show increased collaborative communication and conflict management strategies and decreased aggressive and passive-avoidant styles from pre- to post-intervention relative to controls*.

**H2 (Peer conflict tactics):**  *Students receiving the program will report reduced verbal and physical peer conflict tactics (produced and suffered) from pre- to post-intervention relative to controls. Given the language-based nature of the intervention, effects on physical peer conflict tactics were expected to be weaker and were examined as secondary outcomes*.

**H3 (Bullying-related experiences):**  *Students receiving the program may show reductions in bullying-related experiences (psychological harassment, exclusion, physical harassment, relational harassment) but these outcomes were considered distal and exploratory because bullying-related experiences depend on broader peer-group and classroom-level dynamics beyond individual communication micro-skills. Gender effects were examined as baseline differences and as potential moderators (Time × Gender; Group × Time × Gender) without over-stating causal mechanisms. Age and grade-level composition were not specified as directional moderators a priori; instead, they were treated as contextual features and potential sources of non-equivalence, and age was included in sensitivity analyses*.

## 2. Materials and Methods

### 2.1. Design

We used a quasi-experimental, pretest–posttest design with a non-equivalent control group. The within-participant factor was time (pre vs. post). The between-participant factors were group (intervention vs. control) and gender (boys vs. girls). Dependent variables were the subscale scores from the conflict resolution styles measure, peer conflict tactics measure, and bullying-related experiences measure.

### 2.2. Participants

Participants were recruited from public schools in Pontevedra (Spain). Students were aged 10–13 years (*M* = 11.37, *SD* = 0.79). Because classes were pre-existing, allocation to intervention vs. control followed classroom assignment (non-randomized). The final preprocessed dataset included 133 students. Of these, 89 participants belonged to the experimental group and 44 to the control group. Age differed between groups, with the control group (*M* = 12.02, *SD* = 0.73) being older than the experimental group (*M* = 11.04, *SD* = 0.60), Welch’s *t*(72.61) = 7.68, *p* < 0.001. Because allocation was based on intact classrooms, group membership was also related to grade-level composition. According to the school coding, the experimental group included students from Grades 5 and 6 of Primary Education (n = 30 and n = 59, respectively), whereas the control group included students from Grade 6 of Primary Education and Grade 1 of Secondary Education (n = 15 and n = 29, respectively). Therefore, the age difference between groups may partly reflect grade-level composition and possible educational transition effects (ages are listed in Table 1). This imbalance was considered when interpreting the findings and motivated the age-adjusted sensitivity analyses reported below.

### 2.3. Measures

Three measures were used to assess students’ self-reported conflict resolution styles, peer conflict tactics, and bullying-related experiences.

Conflict resolution styles were assessed using the *Escala de Estilos de Resolución de Conflictos Interpersonales* (MERCI), a 50-item self-report measure rated on a 5-point Likert scale (0 = “It never happens to me/I never do it”; 4 = “It often happens to me/I do it very often”). Items yield three subscales, collaborative style (α reported as 0.84), aggressive style (α = 0.74), and passive-avoidant style (α = 0.80), as reported in prior work (Fariña et al., 2021).

Peer conflict tactics were measured using peer-relevant blocks adapted from the Conflict Tactics framework (CTS-PC approach), indexing verbal behaviors (e.g., insulting, yelling, threatening) and physical behaviors (e.g., slapping, hitting with an object, kicking). For each domain, items distinguished produced behaviors (what the student reports doing) from suffered behaviors (what the student reports receiving from others). Responses were rated 0 = “Never”, 1 = “Sometimes”, and 2 = “Often”. The original Conflict Tactics Scales were developed to assess conflict and aggression in family or intimate-partner contexts, and the Parent–Child Conflict Tactics Scale (CTSPC) specifically assess parents’ tactics when responding to conflict or hostility toward their children (Straus et al., 1998). The original CTSPC includes 22 items organized into three main scales, non-violent discipline, psychological aggression, and physical assault, with reported internal consistency estimates of α = 0.70, α = 0.60, and α = 0.55, respectively. CTSPC—Spanish version has documented psychometric properties in parent–child contexts (Dominguez et al., 2025). The physical assault scale is further differentiated according to severity, and items are answered using frequency categories referring mainly to the previous year. In the present study, selected behaviorally concrete items were adapted to refer to interactions with peers rather than parent–child interactions. Items indexed verbal behaviors (e.g., insulting, yelling, threatening) and physical behaviors (e.g., slapping, hitting with an object, kicking). For each domain, items distinguished produced behaviors (what the student reports doing) from suffered behaviors (what the student reports receiving from others). Responses were rated 0 = “Never”, 1 = “Sometimes”, and 2 = “Often”. Because the original CTSPC was not developed for peer-to-peer conflict, the adapted scores should be interpreted cautiously as indicators of peer-directed verbal and physical conflict tactics. To our knowledge, the peer-adapted version used in this study has not undergone full structural validation, such as confirmatory factor analysis, in this specific format. The adapted item wording is provided in the Supplementary Materials to support transparency and future validation work. Given that the original CTS-PC context is parent–child conflict, we restrict interpretation to peer-directed behaviors and provide the peer-adapted wording and item list in the Supplementary Materials to support transparency and content validity evaluation. Internal consistency estimates in the current sample are reported in Supplementary Table S1.

Bullying-related experiences were assessed using the bullying evaluation scale developed and validated by Arce and colleagues (Arce et al., 2014), which measures multiple subdomains relevant to peer harassment and exclusion. In this paper, we use “bullying-related experiences” as an umbrella label for the scale’s subdomains: psychological harassment, exclusion, physical harassment, and relational harassment. We avoid ambiguous terms such as “damage suffered” unless directly tied to a defined validated score; in this manuscript, all bullying outcomes are reported using the scale’s named subdomains. Internal consistency estimates for all scales and subscales in the present sample are reported in Table S1.

Students also completed a short sociodemographic questionnaire (age, gender, and grade/course level).

### 2.4. Intervention: The Reformulando Program

The *Reformulando* program is a manualized didactic unit designed to promote peaceful coexistence by training pragmatic reformulation and respectful (“friendly”) language in peer conflict contexts (Cuevas-Alonso et al., 2016). The intervention comprised ten structured 50 min sessions delivered during the last two academic terms, implemented during regular class time. Each session included brief instruction, modeled examples, guided practice (role-plays and vignette-based exercises), and reflection/transfer activities. Core components included: identifying communicative intent in conflict, paraphrasing without escalation, acknowledging emotion appropriately, and proposing collaborative next steps. To address reproducibility concerns, a session-by-session outline with learning objectives and concrete examples of reformulation and respectful language scripts is provided in the Supplementary Materials. Implementation was conducted by a trained conflict-resolution professional experienced in delivering the program. The intervention was aligned with socio-emotional learning approaches by emphasizing communication, emotional awareness, perspective-taking, and collaborative problem-solving within everyday peer interactions.

### 2.5. Procedure, Safeguarding, and Ethics

Data were collected before (pretest) and after (posttest) program delivery. Students completed questionnaires individually at school in two assessment waves. Each wave involved one session of approximately 60 min; this duration reflects the full battery (multiple scales plus sociodemographics) and the need to ensure that students understood items, could ask clarification questions, and could pause when needed. Administration was supervised by trained researchers who emphasized voluntariness and the right to skip any question.

Because questionnaires included sensitive content (peer harassment, exclusion, aggression), a safeguarding protocol was implemented. Students were informed that participation was voluntary, that they could stop at any time, and that no negative consequences would follow. If a student appeared distressed, administration was paused; students were offered breaks and the option to discontinue. School counseling staff were available according to the school’s standard student-support procedures, and any disclosure indicating risk or ongoing harm was handled through the school’s established child protection pathway, consistent with institutional policy and ethical standards for research with minors.

The control group followed the usual school curriculum during the intervention period and did not receive *Reformulando* or any alternative socio-emotional, coexistence, or conflict-resolution program. Thus, the control condition should be understood as a business-as-usual comparison group rather than as an active control condition. Both groups completed the same assessment battery at pretest and posttest.

The study received approval from the Ethics Committee of the Faculty of Education and Sports Sciences (University of Vigo). Schools granted institutional permission, and written parental consent was obtained for all participating students; student assent was obtained in age-appropriate form. Copies of the participant information sheet and consent/assent templates, as well as the full item lists used in the assessment battery, are included in the OSF repository to increase transparency.

### 2.6. Data Analysis

Descriptive statistics were computed by group and time for all outcome variables. Missing data were handled at the outcome level. Because the primary analyses focused on pre–post change, participants were included in each analysis only when they had valid scores at both pretest and posttest for the corresponding outcome. Therefore, sample sizes vary across outcomes. The percentage of missing complete pre–post pairs ranged from 0.0% for peer aggression outcomes to 25.6% for the collaborative conflict resolution subscale. Missingness was 0.0% for all verbal and physical peer aggression outcomes, 0.8% for total bullying-related experiences, 11.3–15.8% for bullying-related subdomains, and 18.0–25.6% for MERCI subscales. Given the modest sample size, the quasi-experimental classroom-based design, and the fact that missingness occurred at the outcome level after scale scoring, multiple imputation or FIML was not used as the primary missing-data strategy. Instead, analyses were conducted using available complete pre–post pairs for each outcome, and the varying Ns are reported in the corresponding tables. Detailed missing-data information is reported in Supplementary Table S6. To examine intervention-related change, change scores were calculated as POST minus PRE for each participant. Multivariate analyses of variance (MANOVAs) were then conducted on change scores for three outcome domains: conflict resolution styles, peer conflict tactics, and bullying-related experiences. The main effect of group on change scores was interpreted as the Group × Time effect, indicating whether pre–post change differed between the experimental and control groups. Gender and Group × Gender effects on change scores were interpreted as Gender × Time and Group × Gender × Time interactions, respectively. Follow-up univariate ANOVAs were conducted for each outcome. As a sensitivity analysis, ANCOVAs were also performed, predicting posttest scores from group, gender, and their interaction while controlling for pretest scores. Given the significant age imbalance between groups, an additional set of ANCOVA models was conducted including age as a second covariate. These models predicted posttest scores from pretest score, age, group, gender, and the Group × Gender interaction.

Correlations among the study variables are presented in Figure S1 and were computed using pretest data only, to avoid post-intervention changes influencing the correlation structure. Baseline group differences are reported in Table S4, and the age-adjusted ANCOVA sensitivity models are reported in Table S5. Because students were assigned within pre-existing classroom groups, the assumption of independent observations may be partially compromised. To address this issue, we conducted additional sensitivity analyses with cluster-robust standard errors based on the available classroom-level variable. These models predicted change scores from group, gender, age, and the Group × Gender interaction. Group and gender were effect-coded, and age was centered within each model. The group coefficient therefore represents the adjusted Experimental–Control difference in pre–post change. Because only four clusters were available, these analyses were interpreted as sensitivity checks rather than as definitive replacements for the primary MANOVA/ANCOVA models. The full cluster-adjusted sensitivity results are reported in Supplementary Table S7.

## 3. Results

Descriptive statistics by group and time are presented in Table 2. Because analyses were conducted using outcome-specific complete pre–post pairs, Ns vary across outcomes. Detailed missing-data information is reported in Supplementary Table S6. Correlations among conflict resolution styles, peer conflict tactics, and bullying-related experiences were statistically significant for several pairs of variables (Figure S1), supporting the use of multivariate approaches given the conceptual and empirical relatedness of outcomes. Figure S1 is based on pretest data only.

MANOVAs were conducted on change scores to examine whether pre–post changes differed by group and gender. Thus, the effect of group on change scores was interpreted as the Group × Time effect, the effect of gender as the Gender × Time effect, and the Group × Gender effect as the Group × Gender × Time interaction. Full multivariate statistics are provided in Table 3.

### 3.1. Multivariate Effects

The MANOVA conducted on change scores for conflict resolution styles did not show a significant Group × Time effect, Pillai’s Trace = 0.011, *F*(3, 84) = 0.319, *p* = 0.811, suggesting that changes in collaborative, aggressive, and passive-avoidant styles did not differ significantly between the experimental and control groups. In contrast, the MANOVA for peer conflict tactics showed a significant Group × Time effect, Pillai’s Trace = 0.081, *F*(4, 126) = 2.760, *p* = 0.031, indicating that changes in peer aggression differed between groups. The MANOVA for bullying-related experiences also showed a significant Group × Time effect, Pillai’s Trace = 0.175, *F*(4, 100) = 5.320, *p* < 0.001. In addition, a significant Group × Gender × Time interaction was observed for bullying-related experiences, Pillai’s Trace = 0.139, *F*(4, 100) = 4.040, *p* = 0.004, suggesting that changes in bullying-related outcomes varied as a function of both group and gender.

### 3.2. Univariate Effects

Regarding conflict resolution styles, collaborative style increased from pretest to posttest in the overall sample, ΔM = 5.25, *t*(98) = 3.36, *p* = 0.001, dz = 0.34. However, the Group × Time effect was not significant for collaborative style, F(1, 95) = 1.073, *p* = 0.303, ηp^2^ = 0.011, indicating that the increase was not significantly greater in the experimental group than in the control group. No significant Group × Time effects were found for aggressive style, *F*(1, 105) = 0.103, *p* = 0.749, ηp^2^ = 0.001, or passive-avoidant style, *F*(1, 100) = 0.515, *p* = 0.475, ηp^2^ = 0.005.

Follow-up univariate analyses showed significant Group × Time effects for verbal peer aggression. The experimental group showed a decrease in total verbal aggression from pretest to posttest, whereas the control group showed a slight increase, *F*(1, 129) = 10.998, *p* = 0.001, ηp^2^ = 0.079. Similar effects were observed for verbal aggression produced, *F*(1, 129) = 7.021, *p* = 0.009, ηp^2^ = 0.052, and verbal aggression suffered, *F*(1, 129) = 9.751, *p* = 0.002, ηp^2^ = 0.070. These initial univariate results suggest that the program may be associated with reductions in verbal forms of peer conflict, which are the behaviors most directly targeted by language-based communication training. However, these effects were further examined in sensitivity analyses adjusting for age and classroom clustering. By contrast, Group × Time effects were not significant for physical aggression outcomes, suggesting that physical forms of peer aggression were less sensitive to the intervention during the study period.

Bullying-related experiences decreased substantially from pretest to posttest in both groups. However, the significant Group × Time effects reflected larger reductions in the control group than in the experimental group. This pattern was observed for total bullying-related experiences, F(1, 128) = 13.220, *p* < 0.001, ηp^2^ = 0.094, psychological harassment, F(1, 109) = 20.836, *p* < 0.001, ηp^2^ = 0.160, exclusion, F(1, 114) = 13.971, *p* < 0.001, ηp^2^ = 0.109, physical harassment, F(1, 113) = 15.353, *p* < 0.001, ηp^2^ = 0.119, and relational harassment, F(1, 110) = 4.521, *p* = 0.036, ηp^2^ = 0.040. Because the control group showed higher baseline levels in several bullying-related outcomes, these findings should be interpreted cautiously and may partly reflect regression to the mean or baseline imbalance rather than a stronger control group improvement. Thus, H3 was not supported in a program-specific sense, because reductions in bullying-related experiences were not greater in the experimental group than in the control group.

Detailed univariate results for all outcomes are available in Table S2.

### 3.3. Sensitivity Effects

Sensitivity analyses using ANCOVA were conducted to examine posttest differences between groups while controlling for pretest scores. Full ANCOVA sensitivity analyses adjusted for pretest scores are reported in Table S3. Because the control group was older than the experimental group, additional ANCOVA models were conducted including age as a second covariate. In these age-adjusted models, age was not a significant covariate for any outcome. Adjusted group effects remained significant for collaborative conflict resolution style, *F*(1, 93) = 10.794, *p* = 0.001, ηp^2^ = 0.104; passive-avoidant style, *F*(1, 98) = 8.709, *p* = 0.004, ηp^2^ = 0.082; total verbal peer aggression, *F*(1, 127) = 6.675, *p* = 0.011, ηp^2^ = 0.050; and verbal aggression suffered, *F*(1, 127) = 7.149, *p* = 0.008, ηp^2^ = 0.053. The adjusted group effect for verbal aggression produced was attenuated and did not reach conventional significance after including age, *F*(1, 127) = 2.926, *p* = 0.090, ηp^2^ = 0.023. No significant age-adjusted group effects were observed for physical peer aggression or bullying-related experiences. These findings suggested that the age-adjusted pattern was clearer for total verbal peer aggression and verbal aggression suffered than for verbal aggression produced. However, the classroom-clustered sensitivity analyses reported below further attenuated these effects. Detailed age-adjusted sensitivity effects are reported in Table S5. Additional cluster-adjusted sensitivity analyses were conducted using cluster-robust standard errors based on the available group/class variable. Because only four clusters were available, these analyses were interpreted cautiously. After cluster adjustment, the group effect for total verbal peer aggression was attenuated and reached only marginal significance, *B* = −1.299, SEcluster = 0.430, *t*(3) = −3.017, *p* = 0.057, 95% CI [−2.668, 0.071]. A similar pattern was observed for verbal aggression suffered, *B* = −0.832, SEcluster = 0.293, *t*(3) = −2.843, *p* = 0.066, 95% CI [−1.763, 0.099]. The group effect for verbal aggression produced was not significant, *B* = −0.467, SEcluster = 0.237, *t*(3) = −1.973, *p* = 0.143, 95% CI [−1.220, 0.286]. No cluster-adjusted group effects were observed for physical peer aggression or bullying-related experiences. Although the cluster-adjusted model suggested an effect for the passive-avoidant conflict resolution style, this finding was not consistent with the primary change-score analyses or the age-adjusted interpretation and was therefore not treated as evidence of intervention effectiveness. The full cluster-adjusted sensitivity results are reported in Supplementary Table S7.

## 4. Discussion

This study evaluated *Reformulando*, a school-based educational intervention aimed at fostering indicators of socio-emotional functioning through pragmatic reformulation and respectful language. Overall, the findings suggest a cautious and specific pattern. *Reformulando* was associated with initial reductions in verbal peer aggression, but these effects were attenuated after accounting for age imbalance and classroom clustering. Collaborative conflict management increased over time, although this change did not differ significantly between the experimental and control groups. Bullying-related experiences also decreased during the study period, but these reductions were not specific to the experimental group and should not be interpreted as evidence of program effectiveness.

The pattern of findings is consistent with the premise that communication practices can function as trainable socio-emotional skills, particularly in relation to verbal peer aggression.

A key contribution of the present work is the explicit focus on reformulation as a teachable technique with practical relevance to everyday conflict, extending mediation-informed communication approaches into the school setting. The clearest pattern concerned verbal peer aggression. However, the classroom-adjusted sensitivity analyses showed that the effects for total verbal peer aggression and verbal aggression suffered were attenuated when cluster-robust standard errors were applied. Therefore, these findings should be interpreted as promising but not definitive evidence that the intervention may reduce verbal escalation in peer conflict. Cluster-adjusted sensitivity analyses did not support program-specific effects on physical peer aggression or bullying-related experiences. This reinforces the interpretation that broader forms of harassment, exclusion, and systematic bullying are unlikely to be modified by communication micro-skills alone and may require broader, longer, and multi-component school-level interventions. These results suggest that training communication-based socio-emotional skills may support healthier peer relationships, emotional regulation, and a positive school climate, particularly when integrated within broader school-based prevention strategies.

The findings also support the idea that everyday classroom communication may function as a relevant context for socio-emotional development. From this perspective, interventions targeting how students interpret, verbalize, and negotiate interpersonal conflict may contribute not only to aggression reduction, but also to the promotion of emotionally supportive peer environments and constructive school coexistence.

Prior research has shown that boys tend to be more involved in physical and verbal bullying, whereas girls often show greater involvement in relational forms of peer aggression and victimization (Wang et al., 2009; Marcos et al., 2022). In the present study, gender-related interaction effects were observed for bullying-related experiences, indicating that change patterns varied according to both group and gender. However, given the baseline imbalance between groups and the larger reductions observed in the control group for several bullying-related outcomes, these effects should be interpreted cautiously.

Interpretation should remain cautious. The quasi-experimental design limits causal inference, and reliance on self-report introduces response biases (e.g., social desirability, recall). Correlations among outcomes cannot establish directionality (e.g., whether collaborative style reduces victimization or whether lower victimization enables collaboration). In this sense, the observed improvements may be partially explained by the role of reformulation as a language-mediated reappraisal process through which individuals reinterpret conflict situations in less threatening terms (Rolán, 2021). Future work using randomized designs, longer follow-ups, and multi-informant measures (teacher reports, peer nominations, observational indicators) would clarify mechanisms and durability. In addition, the present findings are consistent with the broader view that language is not merely a conduit but a constitutive component of conflict processing, as linguistic framing influences how conflict is encoded, evaluated, and emotionally experienced (Rolán et al., 2025; Rolán, 2021). Although the present evaluation focused on offline peer interactions, adolescent conflict and victimization increasingly extend to digital contexts; evidence with Spanish adolescents suggests that cybervictimization co-occurs with problematic internet use, reinforcing the value of integrating communication training with digital citizenship and online safety content in future iterations of school-based prevention programs (Marcos et al., 2025). From an educational perspective, these findings reinforce the idea that everyday language use in the classroom is not merely communicative but constitutive of students’ socio-emotional development.

### 4.1. Practical Implications

The present findings have practical implications for school-based prevention and youth support services. First, brief communication-based training can be integrated into ordinary classroom activities without requiring specialized clinical settings. Second, reformulation may offer teachers, school counselors, and conflict-resolution practitioners a concrete micro-skill for helping students reinterpret conflict, reduce verbal escalation, and move toward collaborative responses. Third, because the clearest and most theoretically coherent pattern concerned verbal peer aggression, the program may be particularly useful as a universal or selective prevention strategy for improving everyday peer interaction before conflicts become chronic. Fourth, the ten-session structure could be embedded into existing civic, health education, tutoring, or school coexistence curricula without replacing core academic content, especially if sessions are distributed across regular classroom periods. Finally, the results suggest that school-based programs should monitor different forms of peer aggression and victimization separately rather than treating bullying as a single homogeneous construct. Because the program consists of ten structured 50 min sessions delivered during regular class time, it may be transferable to other school contexts with limited disruption to the ordinary curriculum, provided that facilitators receive basic training in conflict communication and safeguarding procedures. Because school climate involves relational safety, peer relationships, and students’ sense of belonging, brief communication-based interventions may contribute to broader prevention efforts when integrated into whole-school approaches (Thapa et al., 2013; Gaffney et al., 2021).

### 4.2. Limitations and Avenues for Further Research

First, groups were non-randomized because classroom assignments were pre-existing; selection effects cannot be ruled out. Baseline differences between groups were observed for several outcomes, particularly bullying-related experiences, with the control group showing higher pretest scores (Table S4). This imbalance may have contributed to larger reductions in the control group and raises the possibility of regression to the mean. In addition, because the allocation was quasi-experimental, teacher or school-level self-selection cannot be ruled out. For example, classrooms or teachers more motivated to engage with coexistence or conflict-resolution activities may have been more likely to participate in the experimental condition, whereas other classrooms may have remained in the control condition. This potential volunteer or implementation-readiness bias should be considered when interpreting program effects. Second, the assessment battery relied on self-report, which can underestimate or overestimate peer aggression and victimization depending on context and perceived norms. Third, missing data should also be considered when interpreting the results. Although peer aggression outcomes had no missing complete pre–post pairs, missingness was higher for some conflict resolution and bullying-related subscales. The analyses therefore used outcome-specific complete pre–post pairs rather than a single constant sample across all outcomes. This approach preserves the available information for each outcome but may introduce bias if missingness was systematically related to unobserved participant characteristics. Future studies with larger samples should use planned missing-data strategies, such as full-information maximum likelihood or multiple imputation, and should examine the missing-data mechanism more formally. Fourth, the study was conducted in a limited regional context; generalizability to other school systems and cultures remains to be tested. Fifth, some constructs (e.g., peer conflict tactics) were derived from an instrument family originally developed for parent–child contexts. Although the CTSPC and its Spanish version have documented psychometric properties in parent–child contexts (Dominguez et al., 2025), in the present study, selected items were adapted because they described concrete verbal and physical behaviors that could also occur in peer conflict. However, the peer-to-peer version used here has not yet undergone full structural validation. Future studies should examine its factorial structure, measurement invariance, convergent validity, and sensitivity to change in school samples. Alternatively, future evaluations could use instruments specifically developed and validated for peer aggression. Sixth, the control group was older on average than the experimental group, and this difference was related to grade-level composition. Specifically, part of the control group came from Grade 1 of the next educational stage, whereas the experimental group came from Grades 5 and 6. This imbalance is relevant because socio-emotional functioning, peer norms, classroom hierarchies, and conflict-management skills may change rapidly during early adolescence. Although age was included as a covariate in sensitivity analyses, statistical adjustment cannot fully rule out maturation, grade-level, or educational transition effects. Therefore, age-adjusted findings should be interpreted as sensitivity evidence rather than as a substitute for randomized or grade-balanced allocation. Seventh, the classroom-based structure of the intervention should also be considered. Although cluster-adjusted sensitivity analyses were conducted, only four clusters were available in the dataset. Cluster-robust standard errors are unstable with such a small number of clusters, and these analyses should therefore be interpreted as exploratory sensitivity checks. Future studies should use cluster-randomized or stepped-wedge designs with a larger number of classrooms to estimate classroom-level variance and intervention effects more reliably.

Future studies should (a) test the program in randomized or stepped-wedge designs; (b) compare *Reformulando* with an active control condition, such as another socio-emotional learning or school coexistence activity, because the present study used a business-as-usual comparison group and non-specific factors such as attention, novelty, facilitator contact, or increased classroom focus on peer interaction cannot be ruled out; (c) incorporate multi-informant data and objective indicators; (d) examine maintenance at follow-up; and (e) investigate mediators such as emotion recognition, perspective-taking, and pragmatic comprehension to identify mechanisms by which reformulation training impacts peer outcomes. Future research should also examine whether communication-based socio-emotional interventions such as *Reformulando* produce broader educational outcomes, including improvements in classroom climate, emotional wellbeing, school belonging, and interpersonal adjustment.

## 5. Conclusions

In this quasi-experimental pretest–posttest evaluation, *Reformulando* showed a promising but cautious pattern of change in conflict-related outcomes, particularly reductions in verbal peer aggression. However, sensitivity analyses indicated that these effects were attenuated after accounting for age imbalance and classroom clustering, especially for verbal aggression produced. Collaborative conflict management increased over time, although this change did not differ significantly between the experimental and control groups. Bullying-related experiences also decreased during the study period, but these changes were not specific to the experimental group. Overall, the findings suggest that structured communication-based training may help address verbal forms of peer conflict, although stronger evidence from randomized or cluster-randomized studies is needed before drawing firm conclusions about intervention effectiveness.

## Figures and Tables

**Table 1:** Sample distribution by group and gender.

Group	Gender	*N*	Age *M*	Age *SD*
Experimental	Boy	49	10.86	0.65
Experimental	Girl	40	11.28	0.45
Control	Boy	20	12.10	0.79
Control	Girl	24	11.96	0.69
Total	Both	133	11.37	0.79
Experimental	Both	89	11.04	0.60
Control	Both	44	12.02	0.73

**Table 2:** Descriptive statistics by group and time.

	Group	*N* Pairs	Pre *M*	Pre *SD*	Post *M*	Post *SD*	ΔPost–Pre	Δ*SD*
MERCI-1	Exper.	66	52.65	18.86	58.21	17.31	5.56	16.31
	Cont.	33	41.03	20.91	45.67	20.79	4.64	14.11
MERCI-2	Exper.	69	15.71	7.95	14.90	6.86	−0.81	7.66
	Cont.	40	13.73	8.33	13.70	8.89	−0.03	7.31
MERCI-3	Exper.	65	23.63	10.13	23.78	7.61	0.15	9.69
	Cont.	39	18.49	8.24	18.74	6.17	0.26	6.46
VPA (T)	Exper.	89	3.57	2.72	2.37	1.98	−1.20	2.43
	Cont.	44	3.18	2.50	3.52	2.65	0.34	2.32
VPA-P	Exper.	89	1.73	1.57	1.12	1.19	−0.61	1.54
	Cont.	44	1.39	1.28	1.55	1.55	0.16	1.03
VPA-S	Exper.	89	1.84	1.46	1.25	1.23	−0.60	1.25
	Cont.	44	1.80	1.49	1.98	1.44	0.18	1.54
PPA (T)	Exper.	89	1.22	1.79	0.72	1.24	−0.51	1.94
	Cont.	44	0.61	1.19	0.52	0.95	−0.09	1.24
PPA-P	Exper.	89	0.52	0.95	0.29	0.69	−0.22	0.96
	Cont.	44	0.32	0.74	0.32	0.80	0.00	0.84
PPA-S	Exper.	89	0.71	1.12	0.43	0.84	−0.28	1.27
	Cont.	44	0.30	0.59	0.20	0.46	−0.09	0.77
BRE (T)	Exper.	89	22.99	15.20	5.42	9.51	−17.57	16.21
	Cont.	43	31.84	11.75	5.93	7.00	−25.91	9.87
PsyH	Exper.	75	9.96	6.00	1.68	3.37	-8.28	6.46
	Cont.	38	14.63	5.02	2.32	3.43	−12.32	3.70
Exc	Exper.	78	5.24	3.58	1.18	2.19	−4.06	4.10
	Cont.	40	7.30	2.87	1.38	1.92	−5.93	3.13
PhyH	Exper.	80	2.25	1.85	0.19	0.80	−2.06	1.92
	Cont.	38	3.13	0.34	0.26	0.83	−2.87	0.84
RelH	Exper.	74	4.97	3.54	2.03	3.35	−2.95	4.31
	Cont.	38	7.37	3.44	2.16	2.47	−5.21	3.41

*Note*. MERCI-1: collaborative conflict resolution style; MERCI-2: aggressive conflict resolution style; MERCI-3: passive-avoidant conflict resolution style; VPA (T): verbal peer aggression (total); VPA-P: verbal aggression produced; VPA-S: verbal aggression suffered; PPA (T): physical peer aggression (total); PPA-P: physical aggression produced; PPA-S: physical aggression suffered; BRE (T): bullying-related experiences (total); PsyH: psychological harassment; Exc: exclusion; PhyH: physical harassment; RelH: relational harassment; Exper: experimental group; Cont: control group.

**Table 3:** Multivariate tests on change scores.

	*N*	Effect	Pillai’s Trace	*F*	df1	df2	*p*
Conflict resolution styles	90	Group × Time	0.01	0.32	3	84	0.811
	90	Gender × Time	0.04	1.09	3	84	0.358
	90	Group × Gender × Time	0.02	0.56	3	84	0.645
Peer conflict tactics (produced/suffered)	133	Group × Time	0.08	2.76	4	126	0.031
	133	Gender × Time	0.01	0.27	4	126	0.894
	133	Group × Gender × Time	0.02	0.59	4	126	0.669
Bullying-related experiences	107	Group × Time	0.18	5.32	4	100	<0.001
	107	Gender × Time	0.05	1.24	4	100	0.300
	107	Group × Gender × Time	0.14	4.04	4	100	0.004

*Note*. Group × Time interaction tests whether pre–post change differed between the experimental and control groups. Gender × Time tests whether change differed by gender. Group × Gender × Time tests moderated change.

## Data Availability

De-identified data and Supplementary Materials are available at: https://osf.io/k6x2d/ https://osf.io/k6x2d/overview?view_only=484df9e994e443c49e7daf039b637764 (accessed on 15 May 2026).

## Supplementary Materials

The following supporting information can be downloaded at https://www.mdpi.com/article/10.3390/ejihpe16070093/s1, https://osf.io/k6x2d/ https://osf.io/k6x2d/overview?view_only=484df9e994e443c49e7daf039b637764 (accessed on 15 May 2026). Figure S1: Significant correlations (*r* > 0.20; *p* < 0.01) among the variables; Table S1: Internal consistency estimates; Table S2: Univariate change-score analyses; Table S3: ANCOVA sensitivity analyses (posttest adjusted for pretest); Table S4: Baseline differences between groups; Table S5: Age-adjusted ANCOVA sensitivity analyses; Table S6: Missing complete pre–post pairs by outcome; Table S7: Cluster-adjusted sensitivity analyses using cluster-robust standard errors.
