# Supplementary material for: Learning to Reformulate Peer Conflict: Effects of a Language-Based Educational Program on Conflict Management, Peer Interaction, and Bullying-Related Experiences in Early Adolescence
Source: Eur J Investig Health Psychol Educ. 2026 Jun 30;16(7):93. doi: 10.3390/ejihpe16070093 (PMC13408819; doi:10.3390/ejihpe16070093)
Supplement: Supplementary file 1 [file ejihpe-16-00093-s001.zip › ejihpe-4352154-supplementary materials.pdf]

Supplementary Material

Figure S1. *Significant pretest correlations (r > .20; p < .01) among the variables.*

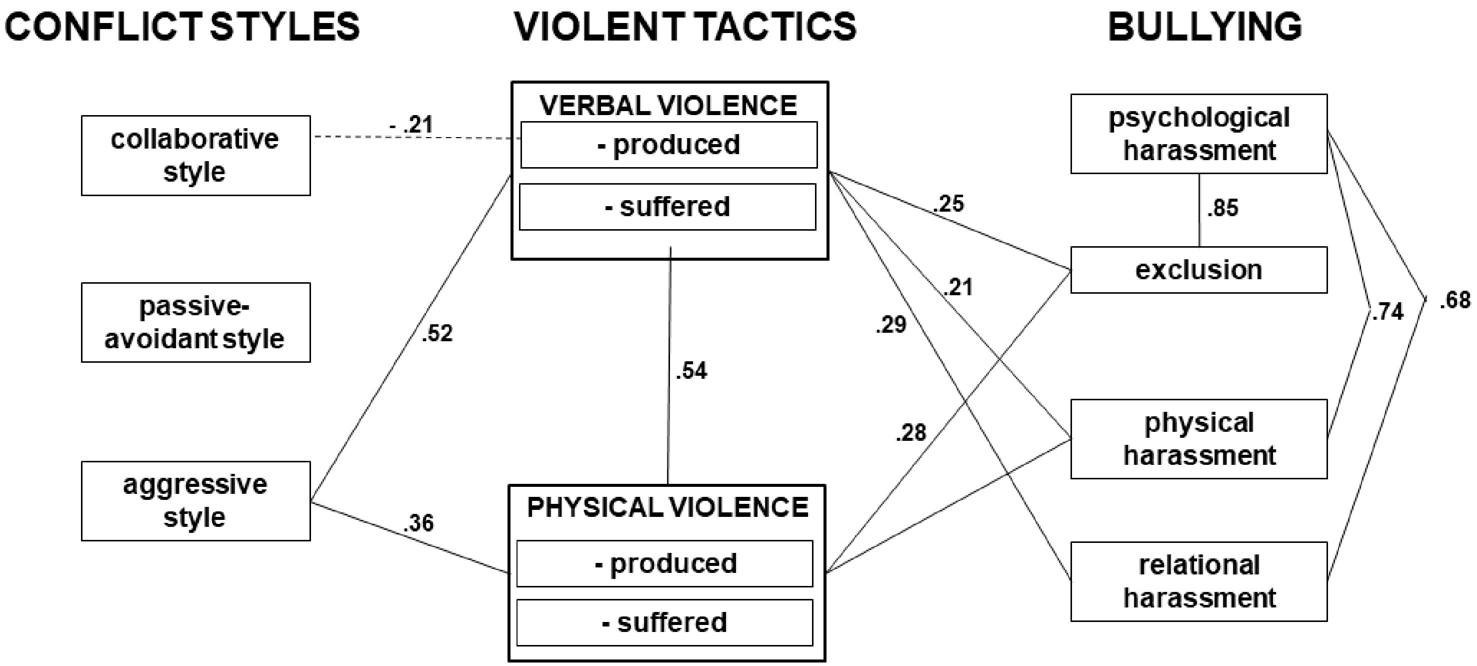

Table S1.

Internal consistency estimates.

	Subscale / score	Number of items	Cronbach's α	Complete N
**MERCI_1**	Collaborative conflict resolution style	20	.94	138
**MERCI_2**	Aggressive conflict resolution style	19	.84	139
**MERCI_3**	Passive-avoidant conflict resolution style	11	.76	148
**Verbal peer aggression**	Verbal peer aggression total	6	.82	154
**Verbal peer aggression**	Verbal aggression produced	3	.74	164
**Verbal peer aggression**	Verbal aggression suffered	3	.70	155
**Physical peer aggression**	Physical peer aggression total	6	.76	138
**Physical peer aggression**	Physical aggression produced	3	.68	161
**Physical peer aggression**	Physical aggression suffered	3	.69	143
**Bullying-related experiences**	Bullying-related experiences total	26	.87	142

Table S2. *Univariate change-score analyses.*

	Experimental Group						Control Group														
		PRE		POST				PRE		POST			Group×Time			Gender×Time			G×Gender×Time		
	N	*M*	*SD*	*M*	*SD*	Δ	*N*	*M*	*SD*	*M*	*SD*	Δ	*F*	*p*	η^2_p	*F*	*p*	η^2_p	*F*	*p*	η^2_p
**MERCI_1**	66	52.65	18.86	58.21	17.31	5.56	33	41.03	20.91	45.67	20.79	4.64	1.07	.303	.01	.09	.761	.00	1.71	.194	.02
**MERCI_2**	69	15.71	7.95	14.90	6.86	-.81	40	13.73	8.33	13.70	8.89	-.03	.10	.749	.00	.55	.461	.01	.02	.902	.00
**MERCI_3**	65	23.63	10.13	23.78	7.61	.15	39	18.49	8.24	18.74	6.17	.26	.51	.475	.01	.00	.952	.00	1.61	.208	.02
**Verbal peer aggression**	89	3.57	2.72	2.37	1.98	-1.20	44	3.18	2.50	3.52	2.65	.34	11.00	.001	.08	.28	.597	.00	1.72	.192	.01
produced	89	1.73	1.57	1.12	1.19	-.61	44	1.39	1.28	1.55	1.55	.16	7.02	.009	.05	.15	.695	.00	.76	.386	.01
suffered	89	1.84	1.46	1.25	1.23	-.60	44	1.80	1.49	1.98	1.44	.18	9.75	.002	.07	.28	.596	.00	2.01	.158	.02
**Physical peer aggression**	89	1.22	1.79	0.72	1.24	-.51	44	.61	1.19	0.52	.95	-.09	1.84	.177	.01	.04	.837	.00	.58	.446	.00
produced	89	.52	.95	0.29	0.69	-.22	44	.32	0.74	0.32	0.80	.00	1.43	.234	.01	.11	.745	.00	.16	.692	.00
suffered	89	.71	1.12	0.43	0.84	-.28	44	.30	0.59	0.20	0.46	-.09	1.25	.266	.01	.35	.555	.00	.74	.391	.01
**Bullying**	89	22.99	15.20	5.42	9.51	-17.57	43	31.84	11.75	5.93	7.00	-25.91	13.22	<.001	.09	.48	.491	.00	4.82	.030	.04
**Psychological harassment**	75	9.96	6.00	1.68	3.37	-8.28	38	14.63	5.02	2.32	3.43	-12.32	20.84	<.001	.16	.69	.408	.01	8.95	.003	.08
**Exclusion**	78	5.24	3.58	1.18	2.19	-4.06	40	7.30	2.87	1.38	1.92	-5.93	13.97	<.001	.11	1.17	.282	.01	8.12	.005	.07
**Physical harassment**	80	2.25	1.85	0.19	0.80	-2.06	38	3.13	0.34	0.26	0.83	-2.87	15.35	<.001	.12	1.68	.198	.01	9.96	.002	.08
**Relational harassment**	74	4.97	3.54	2.03	3.35	-2.95	38	7.37	3.44	2.16	2.47	-5.21	4.52	.036	.04	1.26	.264	.01	.22	.641	.00

Note. MERCI_1: collaborative style; MERCI_2: aggressive style; MERCI_3: passive-avoidant style; verbal peer aggression (total); verbal aggression produced; verbal aggression suffered; physical peer aggression (total); physical aggression produced; physical aggression suffered; bullying-related experiences (total); psychological harassment; exclusion; physical harassment; relational harassment.

Table S3. *ANCOVA sensitivity analyses (posttest adjusted for pretest).*

	PRE-POST				GROUP			GENDER			Group×Gender		
	N	F	p	η^2_p	F	p	η^2_p	F	p	η^2_p	F	p	η^2_p
**MERCI_1**	99	59.41	<.001	.39	8.36	.005	.08	4.39	.039	.04	5.37	.023	.05
**MERCI_2**	109	40.84	<.001	.28	0.11	.739	.00	0.10	.756	.00	0.11	.744	.00
**MERCI_3**	104	31.17	<.001	.24	8.01	.006	.07	1.38	.243	.01	2.62	.109	.03
**Verbal peer aggression**	133	46.86	<.001	.27	10.10	.002	.07	0.93	.336	.01	0.54	.463	.00
Verbal aggression produced	133	43.67	<.001	.25	7.07	.009	.05	1.21	.274	.01	1.06	.305	.01
Verbal aggression suffered	133	44.68	<.001	.26	7.50	.007	.06	0.26	.612	.00	0.07	.795	.00
**Physical peer aggression**	133	4.08	.046	.03	0.10	.755	.00	1.60	.208	.01	0.01	.926	.00
Physical aggression produced	133	14.20	<.001	.10	0.94	.334	.01	3.98	.048	.03	0.26	.612	.00
Physical aggression suffered	133	1.83	.179	.01	1.81	.181	.01	0.01	.906	.00	0.45	.506	.00
**Bullying**	132	9.46	.003	.07	0.10	.755	.00	0.03	.864	.00	0.01	.905	.00
**Psychologica l harassment**	113	12.48	.001	.10	0.95	.331	.01	0.15	.698	.00	1.15	.286	.01
**Exclusion**	118	1.34	.250	.01	0.46	.497	.00	0.71	.400	.01	1.29	.258	.01
**Physical harassment**	118	2.28	.133	.02	2.05	.154	.02	3.55	.062	.03	4.31	.040	.04
**Relational harassment**	112	7.67	.007	.07	0.01	.933	.00	1.26	.265	.01	0.52	.472	.00

Note. MERCI_1: collaborative style; MERCI_2: aggressive style; MERCI_3: passive-avoidant style; verbal peer aggression (total); verbal aggression produced; verbal aggression suffered; physical peer aggression (total); physical aggression produced; physical aggression suffered; bullying-related experiences (total); psychological harassment; exclusion; physical harassment; relational harassment.

Table S4. *Baseline differences between groups.*

	EXPERIMENTAL GROUP			CONTROL GROUP						
	N	*M*	*SD*	*N*	*M*	*SD*	*t*	*df*	*p*	**Hedges' g (Exp-Control)**

**MERCI_1**	82	51.00	18.50	40	43.20	20.77	2.02	70.00	.048	.40
**MERCI_2**	80	16.06	8.28	43	14.72	8.91	.82	80.85	.417	.16
**MERCI_3**	81	23.47	9.71	42	18.29	8.28	3.10	95.52	.003	.56
**Verbal peer aggression**	89	3.57	2.72	44	3.18	2.50	.82	92.64	.412	.15
Verbal aggression produced	89	1.73	1.57	44	1.39	1.28	1.35	103.07	.180	.23
Verbal aggression suffered	89	1.84	1.46	44	1.80	1.49	.17	84.40	.863	.03
**Physical peer aggression**	89	1.22	1.79	44	.61	1.19	2.35	119.98	.021	.38
Physical aggression produced	89	0.52	0.95	44	.32	.74	1.32	107.37	.190	.22
Physical aggression suffered	89	0.71	1.12	44	.30	.59	2.77	130.31	.006	.42
**Bullying**	89	22.99	15.20	44	31.70	11.65	-3.66	108.32	<.001	-.61
**Psychological harassment**	82	10.33	6.12	43	14.37	4.77	-4.07	105.16	<.001	-.71
**Exclusion**	85	5.39	3.57	43	7.26	2.77	-3.26	105.04	.001	-.56
**Physical harassment**	86	2.37	1.93	43	3.12	.32	-3.47	94.22	.001	-.46
**Relational harassment**	82	5.26	3.76	43	7.70	3.87	-3.38	83.30	.001	-.64

Note. MERCI_1: collaborative style; MERCI_2: aggressive style; MERCI_3: passive-avoidant style; verbal peer aggression (total); verbal aggression produced; verbal aggression suffered; physical peer aggression (total); physical aggression produced; physical aggression suffered; bullying-related experiences (total); psychological harassment; exclusion; physical harassment; relational harassment.

Table S5. *Age-adjusted ANCOVA sensitivity analyses.*

	N	Pretest F, p, ηp²	Age F, p, ηp²	Group F, p, ηp²	Gender F, p, ηp²	Group × Gender F, p, ηp²
**MERCI-1**	99	59.37, < .001, .390	2.52, .116, .026	10.79, .001, .104	5.84, .018, .059	7.43, .008, .074
**MERCI-2**	109	40.33, < .001, .281	0.11, .736, .001	0.21, .644, .002	0.12, .725, .001	0.16, .692, .002
**MERCI-3**	104	32.00, < .001, .246	1.07, .303, .011	8.71, .004, .082	1.59, .210, .016	3.27, .073, .032
**VPA (T)**	133	46.33, < .001, .267	0.02, .890, .000	6.67, .011, .050	0.90, .343, .007	0.48, .491, .004
**VPA-P**	133	44.50, < .001, .259	0.89, .346, .007	2.93, .090, .023	1.05, .307, .008	0.66, .419, .005
**VPA-S**	133	44.17, < .001, .258	0.49, .486, .004	7.15, .008, .053	0.30, .582, .002	0.16, .694, .001
**PPA (T)**	133	3.74, .055, .029	1.73, .190, .013	0.20, .659, .002	1.85, .176, .014	0.03, .862, .000
**PPA-P**	133	13.65, < .001, .097	2.61, .109, .020	2.89, .091, .022	4.50, .036, .034	0.70, .405, .005
**PPA-S**	133	1.74, .189, .014	0.21, .649, .002	0.80, .374, .006	0.01, .931, .000	0.32, .575, .002
**BRE (T)**	132	9.38, .003, .069	0.00, .951, .000	0.05, .816, .000	0.03, .861, .000	0.02, .898, .000
**PsyH**	113	12.39, < .001, .104	0.15, .700, .001	0.47, .495, .004	0.14, .712, .001	0.95, .331, .009

	N	Pretest<br>F, p, ηp²	Age<br>F, p, ηp²	Group<br>F, p, ηp²	Gender<br>F, p, ηp²	Group × Gender<br>F, p, ηp²
**Exc**	118	1.27, .262, .011	0.71, .403, .006	1.03, .313, .009	0.84, .362, .007	1.66, .200, .015
**PhyH**	118	2.81, .097, .024	3.60, .060, .031	0.17, .682, .002	3.34, .070, .029	3.07, .082, .027
**RelH**	112	7.53, .007, .066	0.04, .837, .000	0.00, .975, .000	1.19, .278, .011	0.43, .513, .004

Note. MERCI-1 = collaborative conflict resolution style; MERCI-2 = aggressive conflict resolution style; MERCI-3 = passive-avoidant conflict resolution style; VPA (T) = verbal peer aggression total; VPA-P = verbal aggression produced; VPA-S = verbal aggression suffered; PPA (T) = physical peer aggression total; PPA-P = physical aggression produced; PPA-S = physical aggression suffered; BRE (T) = bullying-related experiences total; PsyH = psychological harassment; Exc = exclusion; PhyH = physical harassment; RelH = relational harassment. Models predicted posttest scores from pretest score, age, group, gender, and the Group×Gender interaction.

Table S6. *Missing complete pre–post pairs by outcome.*

Outcome	Valid pre–post pairs	Missing (n)	Missing %
**MERCI total**	125	8	6.0
**MERCI collaborative style**	99	34	25.6
**MERCI aggressive style**	109	24	18.0
**MERCI passive-avoidant style**	104	29	21.8
**Verbal peer aggression total**	133	0	0
**Verbal aggression produced**	133	0	0
**Verbal aggression suffered**	133	0	0
**Physical peer aggression total**	133	0	0
**Physical aggression produced**	133	0	0
**Physical aggression suffered**	133	0	0
**Bullying-related experiences total**	132	1	0.8
**Psychological harassment**	113	20	15.0
**Exclusion**	118	15	11.3
**Physical harassment**	118	15	11.3
**Relational harassment**	112	21	15.8

Table S7.

Cluster-adjusted sensitivity analyses using cluster-robust standard errors.

Outcome	*B* Group	*SE* cluster	*t*(3)	*p*	95% CI
**MERCI collaborative style**	3.664	5.489	0.667	.552	[−13.806, 21.133]
**MERCI aggressive style**	0.548	0.507	1.082	.359	[−1.064, 2.160]
**MERCI passive-avoidant style**	1.200	0.187	6.421	.008	[0.605, 1.795]
**Verbal peer aggression total**	−1.299	0.430	−3.017	.057	[−2.668, 0.071]
**Verbal aggression produced**	−0.467	0.237	−1.973	.143	[−1.220, 0.286]
**Verbal aggression suffered**	−0.832	0.293	−2.843	.066	[−1.763, 0.099]
**Physical peer aggression total**	−0.441	0.385	−1.145	.335	[−1.667, 0.785]
**Physical aggression produced**	−0.315	0.206	−1.529	.224	[−0.970, 0.340]
**Physical aggression suffered**	−0.126	0.259	−0.486	.660	[−0.951, 0.699]
**Bullying-related experiences total**	8.300	5.221	1.590	.210	[−8.315, 24.915]
**Psychological harassment**	3.626	1.698	2.135	.122	[−1.779, 9.031]
**Exclusion**	1.815	1.414	1.284	.289	[−2.685, 6.314]
**Physical harassment**	0.440	0.470	0.937	.418	[−1.056, 1.936]
**Relational harassment**	1.962	1.249	1.571	.214	[−2.012, 5.937]

Note. Models predicted change scores from Group, Gender, Age, and the Group × Gender interaction, using cluster-robust standard errors based on the available classroom-level variable. Group and Gender were effect-coded, and age was centered within each model. The group coefficient represents the adjusted Experimental–Control difference in pre–post change. Because only four clusters were available, these analyses should be interpreted as exploratory sensitivity checks rather than as definitive clustered models.
